# Context-sensitive V1 activity that depends on both coherence and surprise

**DOI:** 10.1016/j.crneur.2026.100168

**Published:** 2026-09-17

**Authors:** Zirui Zhang, Clement Abbatecola, Angus T. Paton, Christiaan N. Levelt, William A. Phillips, Lucy S. Petro, Lars F. Muckli

**Affiliations:** a School of Psychology and Neuroscience, College of Medical, Veterinary and Life Sciences, University of Glasgow, Glasgow, G12 8QB, United Kingdom; b Imaging Centre of Excellence, Queen Elizabeth University Hospital, University of Glasgow, G51 4LB, United Kingdom; c Department of Molecular Visual Plasticity, Netherlands Institute for Neuroscience, Amsterdam, Netherlands; d Department of Molecular and Cellular Neurobiology, Center for Neurogenomics and Cognitive Research, VU University Amsterdam, Amsterdam, Netherlands; e Faculty of Natural Sciences, University of Stirling, FK9 4LA, United Kingdom

**Keywords:** fMRI, Early visual cortex, Predictive processing, Cortical feedback

## Abstract

We investigate how information processing in the early visual cortex, which is tuned to the statistical regularities of sensory inputs, depends critically on the context in which those inputs occur. Neurobiologically inspired computational frameworks of visual processing emphasise functional interactions between higher and lower cortical areas, whereby higher areas with larger receptive fields send top-down (feedback) signals that contextualise bottom-up (feedforward) processing in lower areas. In this human fMRI study, we use visual scenes in which information was partially and temporarily occluded to test cortical processing in the corresponding retinotopic area within V1. By temporally varying the onsets of bottom-up (feedforward) sensory input in one quadrant of the visual field (the target area) and the onset of its surrounding context, we examined how consistent versus inconsistent surrounds influence the processing of bottom-up input to V1 before or after the arrival of feedback signals. We found that responses in the target region depended on both the consistency and the timing of the surround stimuli. Information in the surround increased the V1 BOLD response to the target compared with when the target was presented alone. However, when the target became temporally predictable by a consistent surround shown before the target, the response to the target neither increased nor decreased. We discuss our results in the light of how the neocortex transmits informative and coherent signals.

## Highlights

• Contextual surround information can enhance V1 responses to temporally shifted visual inputs in a target area.
• Contextual feedback to V1 depends on the temporal relationship and consistency of surround and target inputs.
• Surround information increases V1 responses except when predictable targets are preceded by consistent surrounds.

## Graphical abstract

**Figure:**
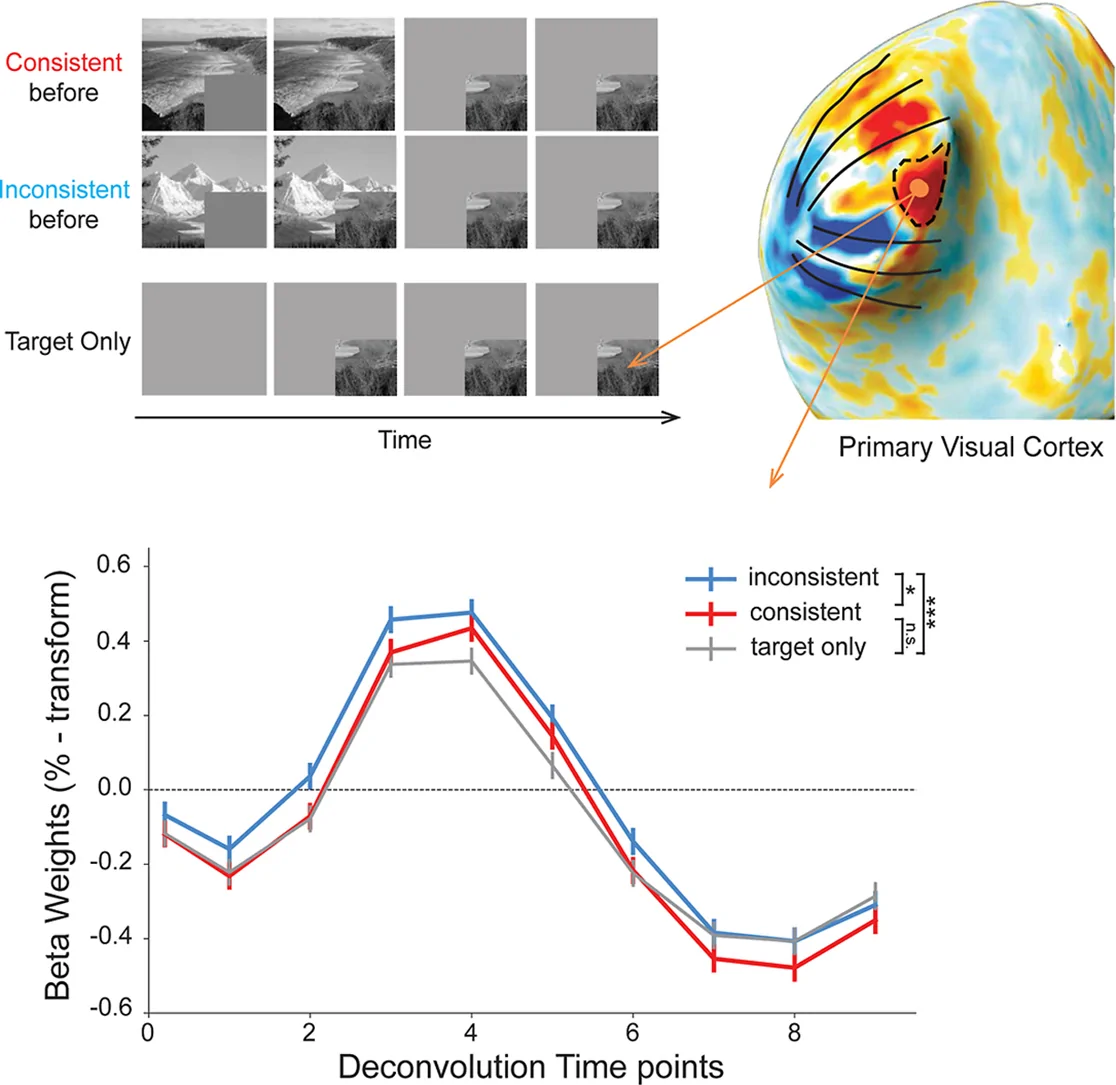

## 1 Introduction

The brain uses predictions generated from prior experience and knowledge to infer external causes of inputs, filter useful information, and make predictions about the future (Teufel and Fletcher, 2020). This relies on comparing neuronal contextual signals with the feedforward flow of sensory information. In the case of visual scene processing, expectations are related to e.g. object features (O'Craven et al., 1999), spatial locations (Petro et al., 2023), gist (Morgan et al., 2019), semantic content (Ortiz-Tudela et al., 2023), and related low and high-level perceptual computations (Svanera et al., 2021), such as the distinction of boundaries between objects (e.g. Self et al., 2019).

Concretely, under the predictive processing framework, activity in a target region of the primary visual area (V1) is under the influence of feedback and lateral information received from the rest of the cortex. This feedback information constitutes a prediction of what feedforward information should be received from the portion of the visual field to which the target cortical region is retinotopically dedicated. From this framework emerges the question of how these two information flows are combined. An important notion here is coherence, defined as the consistency between feedback/predicted and feedforward/sampled information. High coherence means that feedforward information confirms what was predicted. On the other hand, the amount of unpredicted feedforward signal (e.g. when it is inconsistent with predictions) can be quantified as informativeness or surprise (Wibral et al., 2017). Note that “surprise” here is defined from the point of view of a comparison between feedforward and feedback signals by relevant neurons in the target region. This is different from the subjective-level surprise that participants might report behaviourally. Coherence and surprise cannot be both maximised because the most likely interpretation of any data is the least informative, leading to a tension reflected by two apparently opposed bodies of evidence about how top-down expectations influence the processing of bottom-up inputs.

One large body of evidence is linked to the notion of prediction error minimisation, which proposes that during perception, the top-down information flow predicting sensory inputs is confronted with bottom-up information that either confirms our predictions or updates our internal models by computing residual differences between predicted and actual sensory inputs. This results in an increased activation if top-down and bottom-up inputs do not match, related to error signalling, i.e. if predictions of sensory inputs are contradicted by actual sensory inputs. This idea resonates with a range of phenomena that depend on changes, either across space (e.g., as in lateral inhibition), across time (e.g., as in mismatch negativity), or both (e.g., apparent motion; Alink et al., 2010; Edwards et al., 2017). A related hypothesis is that temporally predictable feedforward inputs are ‘explained away’ by top-down predictions. However, recent neuronal recordings in mice and monkeys raise questions on whether neurons in the sensory cortex do compute the difference between actual and predicted inputs (i.e. perform subtractive prediction error coding), at least for layer 2/3 neurons using oddball paradigms to assess predictions (Westerberg et al., 2025; Furutachi et al., 2024; see also Marvan and Phillips, 2024). Instead, prediction error signals in sensory areas might reflect a stimulus-specific amplification of unexpected inputs (Furutachi et al., 2024). In line with these more recent empirical findings, another body of evidence emphasises the amplification of mutually predictive consistent signals in cooperative context-sensitive cognitive computation, which is cooperative to the extent that it drives the signal out of the noise by prioritising the transmission of information coherently related to the context within which it occurs (Phillips, 2017, 2023). This idea is rooted in well-established phenomena under which more probable signals are enhanced in many conditions, such as in figural integration, contextual disambiguation, confirmation bias, change blindness and dissonance reduction (Phillips, 2023). In this framework, information from internal sources, including feedback, provides the context within which feedforward transmission flow is guided toward the amplification of relevant and useful information while attenuating transmission of currently irrelevant information.

Combining coherence and informativeness is possible mathematically (Kay and Phillips, 2020) and evidenced at the cellular level by both multisite patch-clamping in rodent S1 (Kay et al., 2022) and by high-resolution computational modelling of layer 5 pyramidal cells (Graham et al., 2025). Here, we explored the effects of both coherence and informativeness using a partial visual occlusion approach in which a mask covers the lower right quadrant of scene images. Occlusion of a visual segment allows us to isolate feedback signals in the retinotopic visual cortex processing the occluded image region. Human brain imaging in the occluded region has previously revealed that the region of non-stimulated V1 carries information about the surrounding scene, implying contextual information is fed back to cortical areas without feedforward inputs (Smith and Muckli, 2010; Muckli et al., 2015, 2023; Revina et al., 2018; Morgan et al., 2019; Petro et al., 2023; Lazarova et al., 2025). Animal experiments in monkeys (Papale et al., 2023) and mice (Seignette et al., 2024) support this interpretation. Here, we introduce a variant of this occlusion paradigm to investigate if and how top-down expectations of scene features influence the processing of bottom-up inputs which either confirm or violate these top-down expectations: instead of a complete occlusion of the lower right quadrant, we temporally offset the arrival of information to this target region of V1.

Using this occlusion paradigm, prediction error minimisation would imply that, given a surround, the response to an inconsistent target should increase compared to a consistent target, related to the generation of prediction errors. Furthermore, presenting a consistent surround before the target should preempt the appearance of the target, resulting in a reduction of neuronal activity to the target when it appears (i.e. because it is explained away), compared to showing the target only. On the other hand, informativeness maximisation would result in an amplification of the response to target features made more relevant by a consistent surround compared to showing the target only. See Fig. 1 for an illustration of these predictions.

**Fig. 1:**
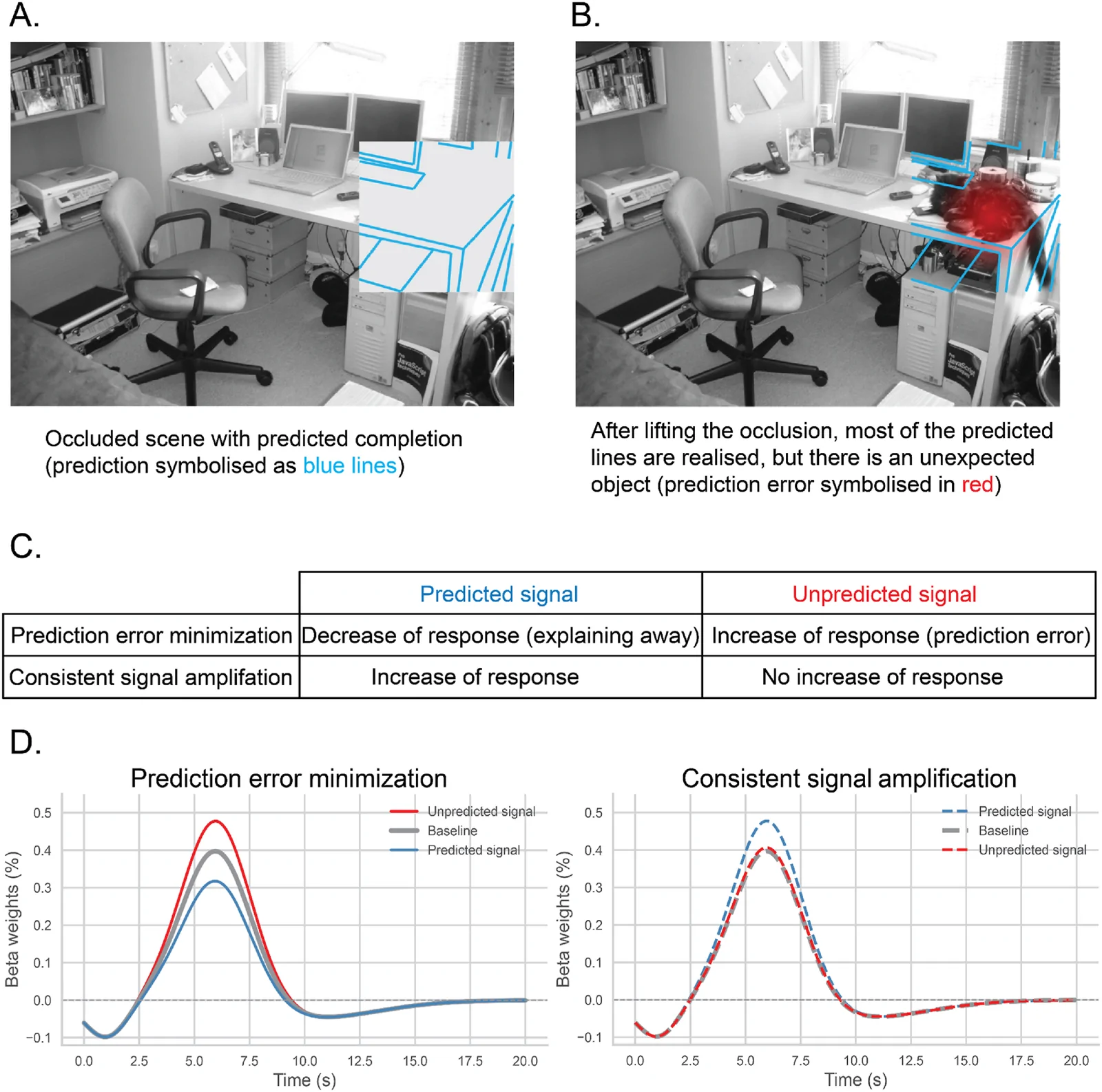
Predictive Processing Theories. A) Occluded scene prediction using line drawing. B) Interaction between unexpected objects and the predicted lines that guide scene interpretation. C) Comparison of predicted and unpredicted BOLD signals, as interpreted by theories of prediction error minimisation and consistent signal amplification. D) BOLD signal simulations demonstrate neural activity patterns under two theoretical frameworks. Left panel: prediction error minimisation, showing reduced activity for minimised discrepancies. Right panel: consistent signal amplification, showing enhanced activity for reinforced predictions. Baseline: response to a target presented in the absence of any surrounding contextual information. Unpredicted signal: response generated when the surrounding contextual information is inconsistent with baseline. Predicted signal: response generated when the surrounding contextual information is consistent with baseline (supporting expectations).

## 2 Results

Twenty-three healthy participants successfully completed the 3T fMRI experiment and were included in the final analysis. The following results are based on this final sample.

We divided the experimental stimuli into a ‘Surround’ (three-quarters of the scene, excluding the lower right quadrant) and a ‘Target’ (the lower right quadrant), Fig. 2 illustrates the experimental design. We modified the temporal order (Surround presented before or after the Target) and the contextual consistency (matching or non-matching scene content between Surround and Target). Finally, we also included a Target-only condition, resulting in five conditions. Each 2.6 s trial presented the Target between 800 and 1800 ms, with Surrounds shown either from 0 to 1000 ms (“before”) or 1600-2600 ms (“after”), resulting in 800 ms between the apparition of the first and second element in all trials, and 200 ms of overlap. We defined individual regions of interest (ROIs) in V1 via retinotopic mapping and GLM contrasts (“target > border” and “target > surround”) representing the lower right target area without involving border and surround voxels. We applied a ROI-wise deconvolution GLM (20 time points) to estimate haemodynamic responses for each condition, generating beta-weighted amplitudes across time. We focused our analyses on peak response points (D3-D6) to test contextual consistency effects on univariate BOLD responses in the V1 Target region.

**Fig. 2:**
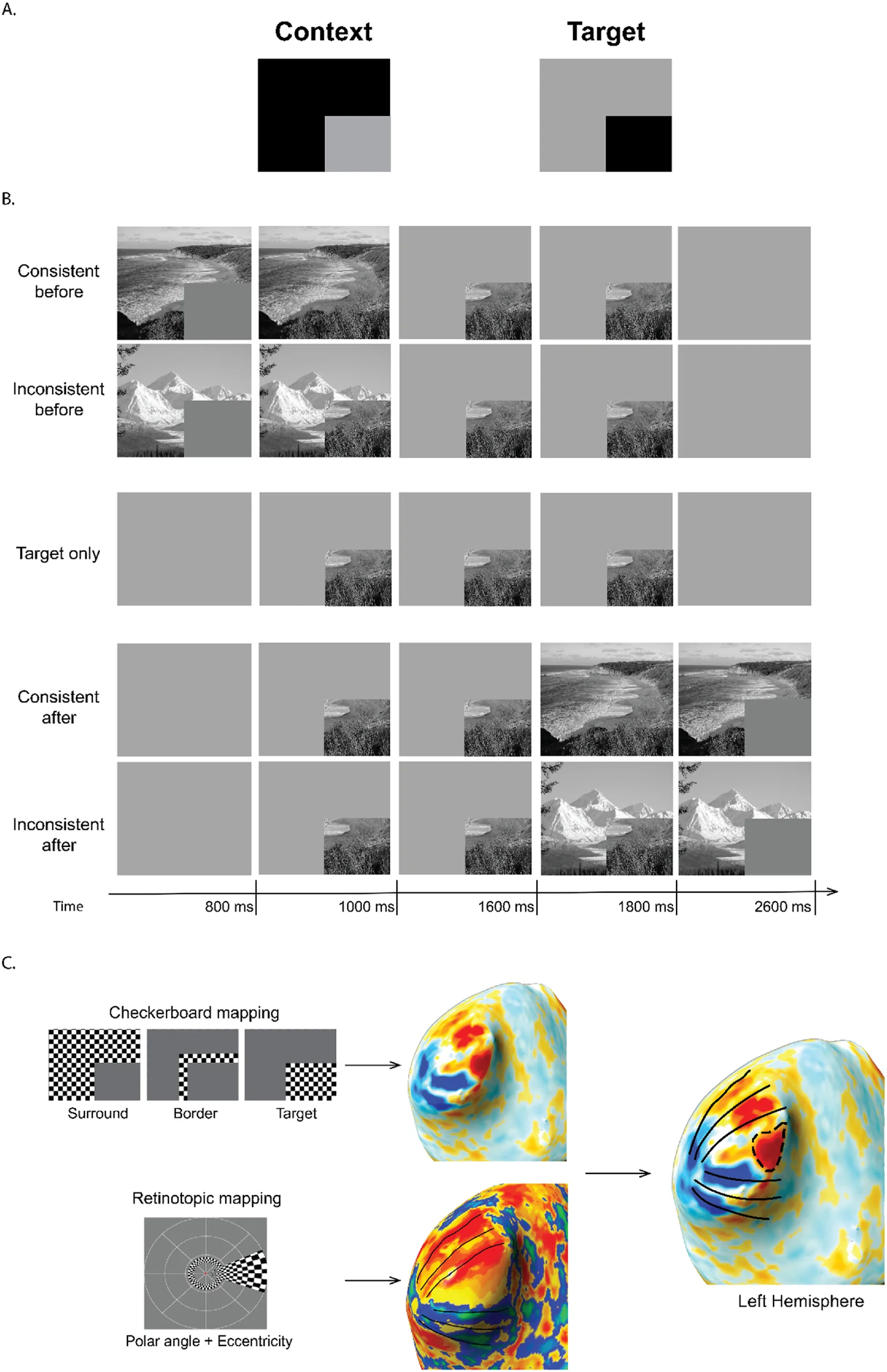
Experimental trials and retinotopic mapping procedures. A) We distinguish two parts of our image stimuli: the ‘Surround’ and the ‘Target’. The Surround refers to the three-quarters of the scene that does not cover the lower right image quadrant (shown in black on the left). The Target refers to the lower right quadrant (shown in black on the right), which either contains scene information (as in the ‘Target only’ condition) or contains a grey occluder. B) The 5 experimental conditions, showing consistent or inconsistent contextual information before or after the presentation of the Target, and one baseline condition with only Target information. C) Checkerboard mapping trials define the target corner region retinotopically in V1: Surround (left), Border (middle), and Target (right). GLM for the contrast: Target - border - surround to define the region of interest. Retinotopic mapping: a combination of polar angle and eccentricity maps the early visual cortex V1-V3 using high-contrast flickering checkerboards, expanding and rotating presentation. The left-hemisphere occipital brain image shows the representation of the Target region (Target - Border - Surround) in V1 (dashed line area).

### 2.1 Top-down contextual information contributes BOLD signal to bottom-up information

First, we tested if adding contextual information induced stronger V1 activity in the region of V1 that processes the Target (i.e. the lower right image quadrant) compared to when there was no contextual information available. Classic theories of vision would not necessarily predict that it would, under the assumption that V1 neurons respond most strongly to driving feedforward inputs and are less affected by modulatory top-down signals. However, the BOLD signal is sensitive to summed postsynaptic membrane potential fluctuations of the dendrites of pyramidal neurons, and therefore captures feedback signals (Larkum et al., 2018; Logothetis, 2008; Logothetis et al., 2001; Viswanathan and Freeman, 2007). We ran GLM contrasts over the peak of the BOLD signal to compare activity in the region of V1 responding to the Target, when only the Target was present versus when there was also any contextual information in the surrounding scene image. We found that the presence of contextual feedback signals (combined here across all 4 conditions vs Target Only) increases the BOLD response in the Target region of V1 compared to when there was no contextual information available, t(22) = 4.703, pFDR = 0.000018, Cohen's d = 0.98 (Fig. 3).

**Fig. 3:**
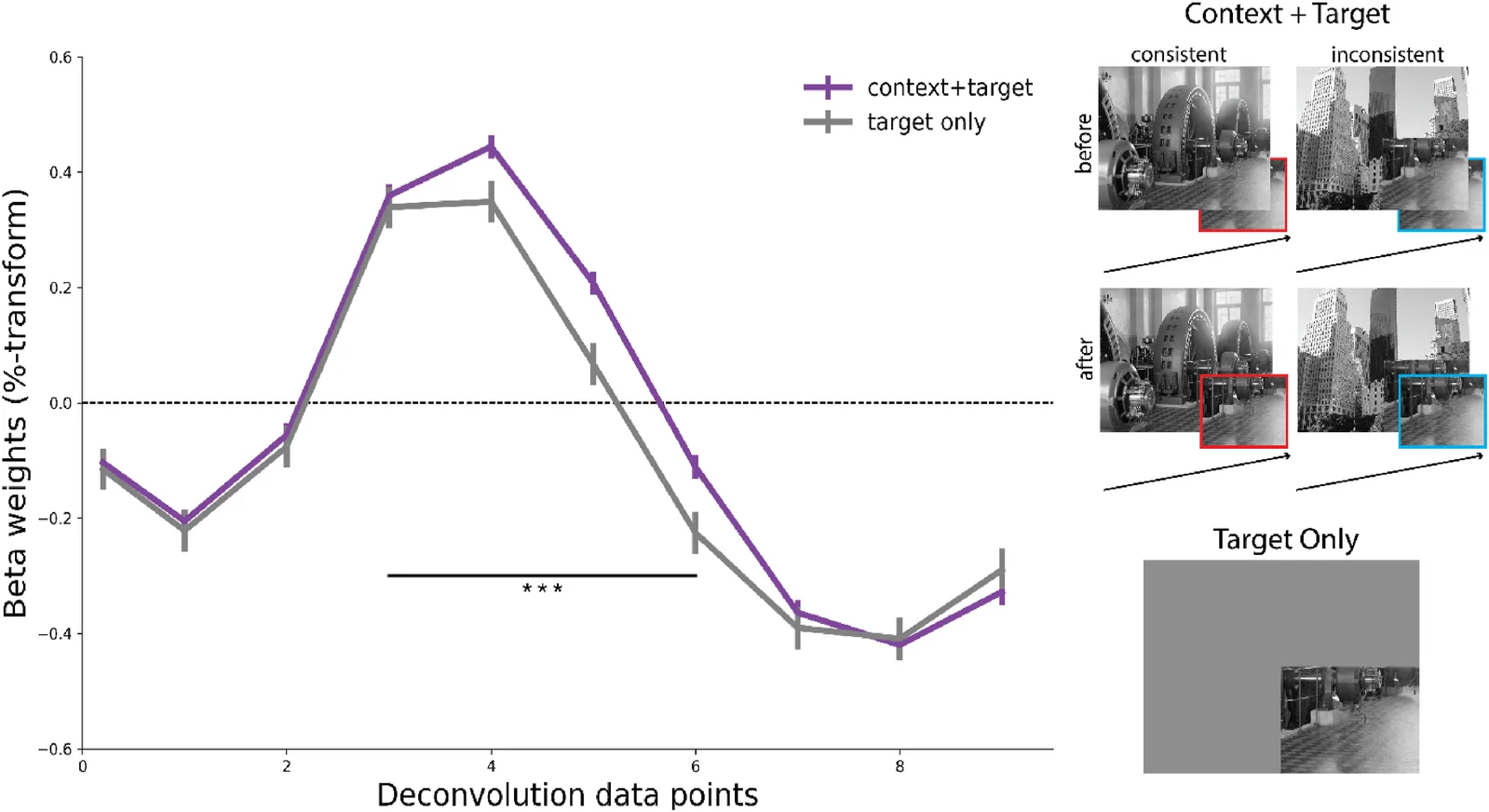
V1 BOLD responses to contextual information plus Target vs Target only, measured in the region of V1 processing the Target. Deconvolution analysis results in the region of interest in V1 that responds to the lower right image quadrant. In all instances, there is bottom-up information available, but in the Context + Target conditions, there is additional contextual information available in the surround to influence processing (on the top right, the colour and position of the Target frame, behind or in front of the Context, indicate consistency and timing). Hence, identical sensory inputs are processed differently depending on the Surround, and this difference is detectable in the BOLD signal. Error bars denote the standard error of the mean (SEM). Stars (*) indicate significant differences from the target-only condition at deconvolution time points D3, D4, D5, D6. *p < 0.05, **p < 0.01, ***p < 0.001 (FDR-corrected).

### 2.2 Contextual feedback signals carry consistency-specific expectations

Next, we investigated how the response to bottom-up activation in the Target region of V1 was influenced by a surround that was either consistent or inconsistent with the target. Compared to the Target only condition, we found significantly higher activity when the Target was preceded by inconsistent contextual information (inconsistent before > Target only, t(22) = 4.703, pFDR < 0.0001, Cohen's d = 0.98). However, we found no significant difference between the Target only and consistent before conditions (consistent before > Target only, t(22) = 2.084, pFDR = 0.11, Cohen's d = 0.43, Fig. 4 top row), suggesting that bottom-up activation is not significantly affected by consistent preceding top-down information, i.e. when the target is made temporally predictable by a consistent surround, the response to the target does not change. Furthermore, there was a significant difference when directly comparing Consistent before vs Inconsistent before (inconsistent before > consistent before, t(22) = 2.585, pFDR < 0.05, Cohen's d​ = 0.54), Fig. 5 left. In sum, inconsistent surrounds increase V1 BOLD response to the target.

**Fig. 4:**
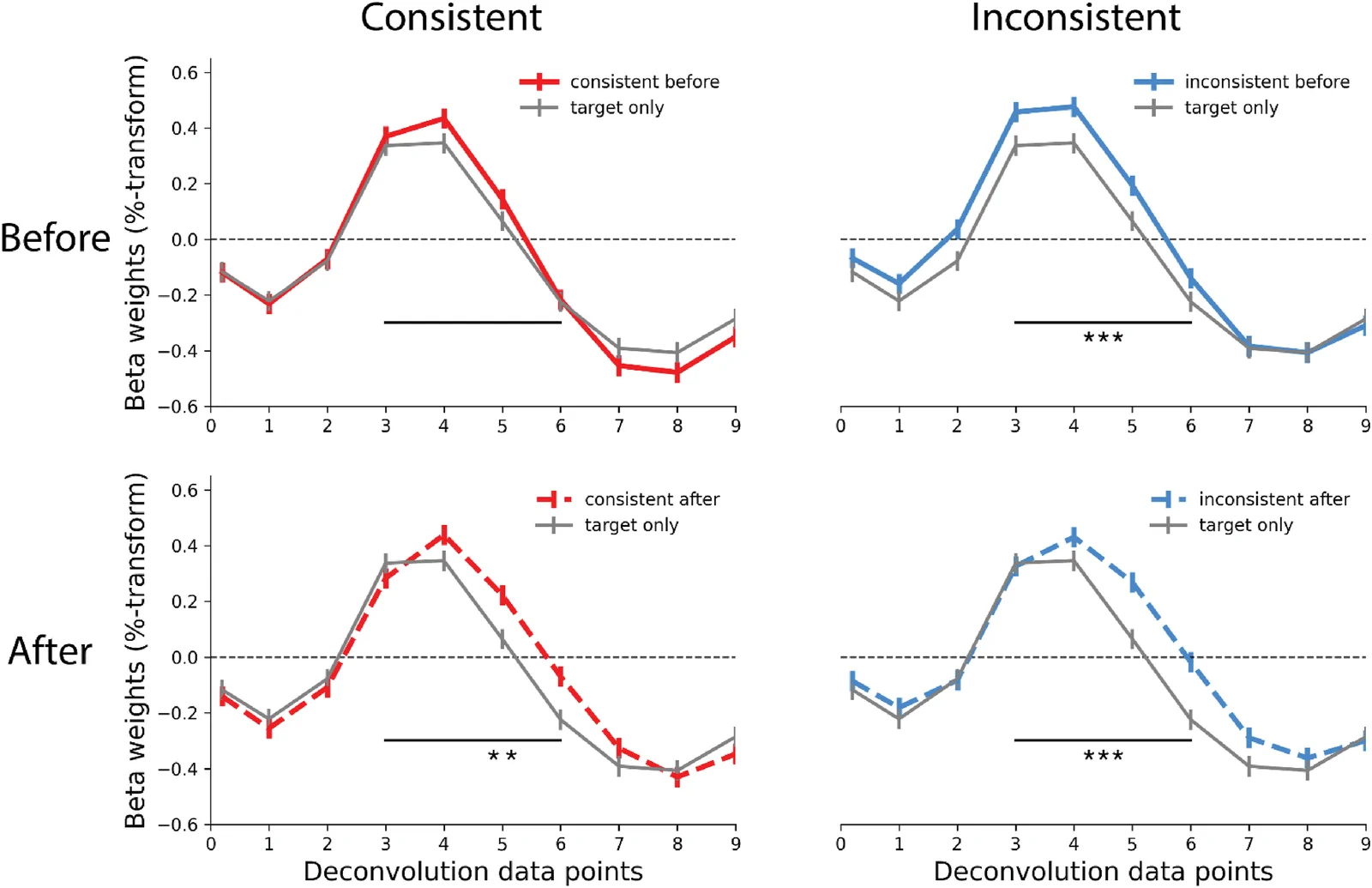
V1 BOLD responses to 4 conditions of contextual information with Target vs Target only, measured in the region of V1 processing the Target. Deconvolution analysis results in the region of interest in V1 responding to the Target (grey), and when there was also consistent (red, full line) or inconsistent (blue, full line) contextual information presented *prior* to the Target being shown (top row), as well as when there was also consistent (red, dotted line) and inconsistent (blue, dotted line) contextual information presented *after* showing the Target (bottom row). Error bars denote the standard error of the mean (SEM). Stars (*) indicate a significant difference between each condition and target only at deconvolution points D3, D4, D5, D6. *p < 0.05, **p < 0.01, ***p < 0.001 (FDR-corrected).

**Fig. 5:**
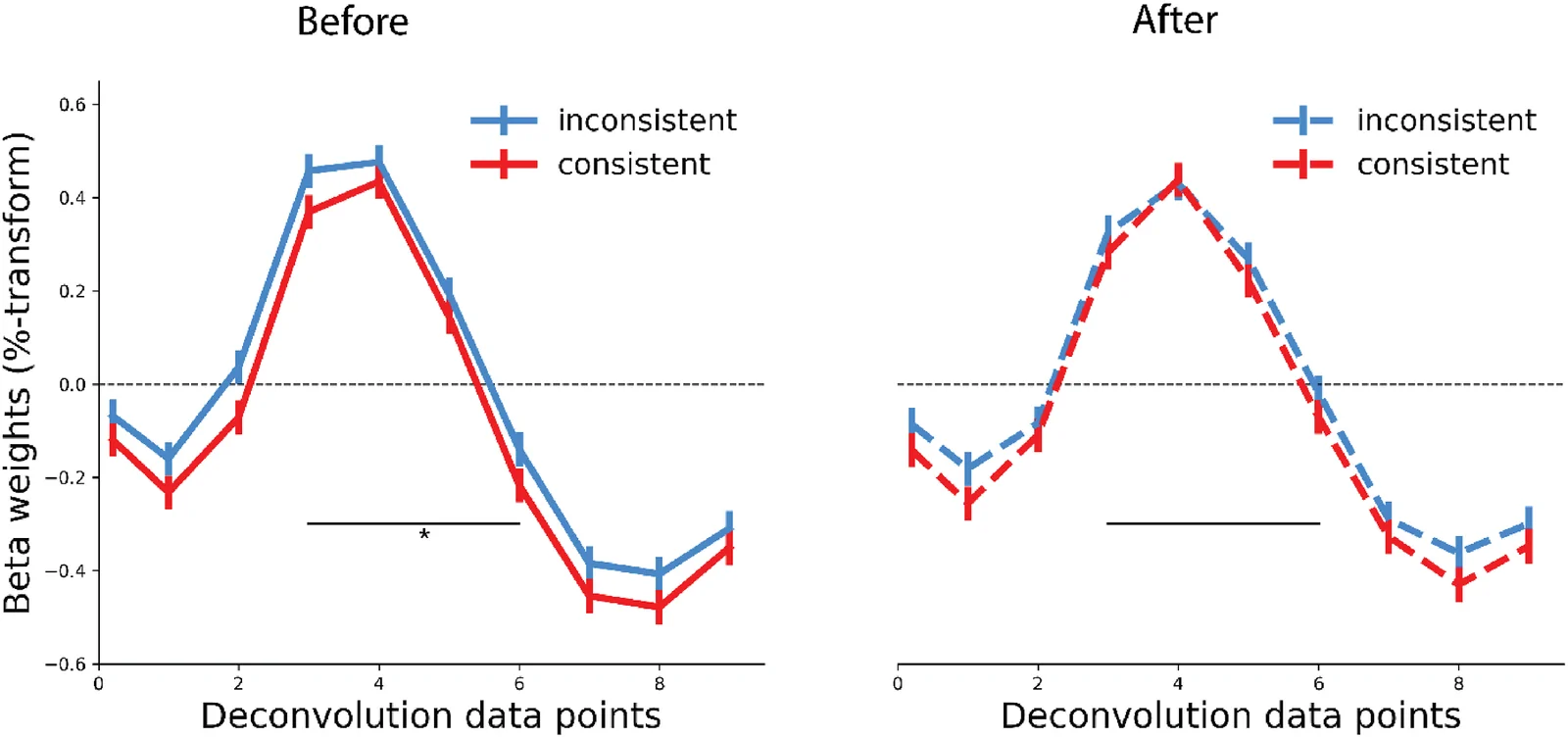
Deconvolution analysis results in the region of interest in V1 show the average BOLD signal changes across 4 sub-conditions: consistent before vs. inconsistent before (left), consistent after vs. inconsistent after (right). Error bars denote the standard error of the mean (SEM). (*) indicate significant differences from the target-only condition at deconvolution time points D3, D4, D5, D6. *p < 0.05, **p < 0.01, ***p < 0.001 (FDR-corrected).

### 2.3 Late contextual feedback signals induce higher BOLD activity regardless of consistency with feedforward information

Our main conditions of interest were the presentation of consistent and inconsistent contextual information that rendered the subsequent bottom-up scene information more or less predictable in a trial based on both general previous experiences and repetition during the scanning session. As a control, we also looked at the effect of reversing the causality by displaying the Target first before consistent or inconsistent Surround information. We found that this unexpected later contextual information increased the response to the bottom-up Target compared to the Target only condition, which was true no matter whether this information was consistent with the Target (consistent after > Target only, t(22) = 3.523, p < 0.01, Cohen's d = 0.73) or not (inconsistent after > Target only, t(22) = 4.859, p < 0.0001, Cohen's d = 1.01, Fig. 4 bottom row). Further, there was no significant difference comparing the response to the Target when it was followed by either consistent or inconsistent contextual information (inconsistent after > consistent after, t(22) = 1.340, pFDR = 0.54, Cohen's d = 0.28), Fig. 5 right.

## 3 Discussion

We have previously shown that non-stimulated regions of V1 receive scene-specific information from the surround, including contextual information that is fed back or laterally transmitted to cortical areas with or without feedforward inputs (Smith and Muckli, 2010; Muckli et al., 2015; Revina et al., 2018; Morgan et al., 2019; Petro et al., 2023; Bennett et al., 2025). The cross-species replications of our human visual occlusion studies suggest that the contextual signals we observe with fMRI are indeed driven by cortical feedback signals and not laterally transmitted information in V1 (Papale et al., 2023; Seignette et al., 2024). Here, we investigated a complementary but different question: how do such top-down expectations influence the processing of bottom-up inputs that either confirm or violate expectations based on the surround? Although previous work has shown that spatial frequency information can be decoded from the early visual cortex (Revina et al., 2018), this study controlled the low-level features, including spatial frequency (see Supplementary Fig. S3), between image categories. This suggests that the observed effects are not driven solely by low-level image properties. Instead, they point to a more abstract form of contextual representation in V1, likely mediated by feedback from higher-order areas, which modulates early visual processing beyond the immediate sensory input.

We found that the response in the target region depends on the consistency and timing of surround stimulation. Information in the surround increases V1 BOLD responses to the target compared to when the target is presented alone, highlighting that feedback processing plays a role even when a feedforward signal is already present. Furthermore, among the four conditions where we presented both target and surround, the only one which does not show this increase is when a consistent surround precedes the target. In that case, the response is not significantly different from presenting the target only, and it is significantly lower than when presenting an inconsistent surround before the target. The comparison between the effect of presenting a context before vs after the target illustrates that consistency only matters when the context precedes the target, supporting our interpretation that predictive top-down signals influence early visual processing.

We wanted in particular to test two concepts of predictive processing against each other: (1) prediction error minimisation, under which inconsistent signals are amplified while consistent signals are explained away, and (2) consistent signal amplification (Fig. 1A). The pattern of our results is challenging to both in their strictest forms, as (1) while we found that inconsistent surrounds induce larger responses to the target compared to the target only, we did not find a decrease of response in the consistent before condition compared to target only (i.e. no explaining away) and both consistent and inconsistent after conditions were similarly increased (i.e. surround effect was not dependent on consistency in these cases), and (2) while we found an increase of response to the target when a consistent surround was presented after it, we found no such increase in the consistent before condition and an increase in both inconsistent conditions. We will now discuss how the two stories may be revised to accommodate our results.

Concerning error minimisation, the first point to consider is that, when presenting the target before surround, the switch between no prediction (nothing in the surround) and any prediction (something in the surround) is in itself a form of surprise, or a change in gist and an indication that the internal models need updating, which could explain the increased response in these two cases. The fact that this increase is not lower when the new surrounding information is consistent with the target could show that these contextual signals prepare for future processing, but are not necessarily taken into account for processing already strong enough feedforward evidence. The lack of response decrease following an increase in predictability, which has also been shown by den Ouden et al. (2024), may be explained in terms of residual surprise. In the consistent before condition, while some information from the target area is correctly predicted, this prediction might not be an eidetic representation of the scene in all of its details, therefore, there will always be a residual amount of unaccounted for information/surprise. This balance between prediction and surprise would be, in the end, comparably surprising to the appearance of the target with no context. Finally, other implementations, such as the free energy principle (Marvan and Phillips, 2024; Phillips et al., 2025), do not necessarily depend upon error coding. Prediction error minimisation is often understood as the subtractive process of comparing descending predictions with ascending sensory inputs, with the difference signal being transmitted up the cortical hierarchy. However, the detection of mismatches or errors, which is highly informative, might be implemented in different ways (Seignette et al., 2024; Marvan and Phillips, 2024; Spratling, 2008), whilst still maintaining the principle of reducing free energy.

Concerning consistent signal amplification, a critical issue is understanding the principles and mechanisms underlying the way in which the thalamocortical system reconciles the conflicting tendencies of amplifying signals because they are coherently related to the context (and therefore less informative), and amplifying them because they are highly informative (here in the case of the inconsistent surrounds). A potential solution to dissociate the two cases would be that the amplifying effects of mutual predictability depend on activities being otherwise sub-maximal. In other words, to see the effects of contextual disambiguation, there must be some ambiguity to be disambiguated. Further developments of the paradigm, with e.g. an increase of feedforward ambiguity, may help shed more light in that direction.

The partial visual occlusion paradigm used here has created surprisingly consistent results across species so far (Papale et al., 2023; Seignette et al., 2024), and further multi-species, multiscale, multi-method research will help to separate how top-down feedback and feedforward input are compared to create stable internal models, disambiguate models, and respond to inconsistencies effectively.

## 4 Limitations of the study

First, although our analyses focus on primary visual cortex (V1), contextual and predictive processing are not limited to early visual areas. Feedback signals are thought to originate from higher-order cortical regions, including parietal, temporal, and prefrontal cortices, which contribute to amodal completion, memory retrieval, and predictive inference (Gilbert and Li, 2013; Muckli and Petro, 2013; Petro et al., 2014; Roelfsema and de Lange, 2016). While our findings demonstrate consistency effects within V1, the broader network dynamics underlying these signals remain unresolved. Future work combining laminar imaging in V1 with simultaneous measurements in higher-order visual and associative regions would help clarify the hierarchical origin and propagation of contextual feedback signals.

Second, although the experimental design was not explicitly aimed at manipulating attention, attentional modulation may have influenced the observed effects. Attention is known to modulate activity across cortical layers and may interact with predictive or contextual processing. In particular, top-down attention could share partially overlapping neural mechanisms (Summerfield and Egner, 2009; Kok et al., 2012). While task demands were controlled and kept consistent across conditions, we cannot fully exclude the possibility that differential attention allocation contributed to the observed differences in brain activity.

Third, the pattern of results may depend on the temporal interval between the presentation of contextual information and the target. Predictive processing in the visual cortex is time-dependent, and different delays between predictive cues and sensory input can alter whether predictions manifest as response suppression or signal amplification (Nara et al., 2021). Although we designed the timing here to balance experimental constraints and BOLD responses, future studies that systematically manipulate these intervals will be important for exploring how temporal dynamics shape predictive modulation in the early visual cortex.

## CRediT authorship contribution statement

Conceptualisation, Z.Z., C.A., L.S.*P. and* L.F.M.; Methodology, Z.Z., C.A., L.S.*P. and* L.F.M.; Data collection, Z.Z.; Data analysis, Z.Z, C.A and A.T.P.; Writing—original draft, Z.Z., C.A. and L.S.P.; Writing-review & editing, C.A, L.S.P., W.A.P., C.L and L.F. M.; Funding acquisition, L.S.P and L.F.M.; Resources, L.S.P and L.F.M.; Supervision, L.S.P and L.F.M.

## Declaration of generative AI and AI-assisted technologies

During the preparation of this work, the authors used ChatGPT (OpenAI) to assist with editing grammar and vocabulary and with the partial implementation of data analysis code (Python). After using this tool, the authors reviewed and edited the content as needed and take full responsibility for the publication's content.

## Declaration of competing interests

The authors declare that they have no known competing financial interests or personal relationships that could have appeared to influence the work reported in this paper.

## Data Availability

The code to run the experiment, plot, and image feature test, part of the analysis, is available on https://github.com/ZZRdpgyg/Timing-Project-. MRI data published on OSF https://doi.org/10.17605/OSF.IO/GPEVJ.

## METHODS

### Participants

Twenty-eight healthy participants from the University of Glasgow took part in the experiment (7 males, aged 18-51 years, mean = 26.21 years). We paid participants for their participation, and they provided informed written consent. The local ethics committee at the University of Glasgow approved the experiment (#200220003). We excluded five participants; two participants were short-sighted (>−6 diopters, which exceeded our ability to correct). One participant did not follow the task instructions, and for one participant, we were unable to define the region of interest. Additionally, one participant was excluded due to unusually noisy BOLD estimates in the V1 ROI. Noise level was assessed using the within-participant standard error of the ROI-averaged deconvolved BOLD response, expressed as percent signal change, computed across trials within each condition. This participant showed standard error values exceeding 0.7 across all conditions, whereas all other participants had values below 0.3, indicating substantially higher signal variability. Therefore, we report results from 23 subjects (5 males, aged 18-51 years, mean = 26.34 years).

### Scene Stimuli

We selected ten real-world scenes from five categories from the Scene Classification Project Upright versus Inverted scene Database (http://vision.stanford.edu/projects/sceneclassification/resources.html; Walther et al., 2009) to induce strong associations between top-down contextual and bottom-up feedforward information (Bar, 2004). We presented these images in grayscale and matched global luminance. We tested low-level image features (luminance, contrast, low-level gist, and spatial frequency) between image categories using the GIST descriptor implemented in Python OpenCV (Bradski, 2000) and MATLAB - Natural Image Statistical Toolbox (Hyvärinen et al., 2009). GIST features were extracted by applying a bank of 32 Gabor filters to each image, consisting of eight orientations (evenly spaced from 0 to π) and four spatial frequencies (5, 7, 9, and 11 pixels). We then performed pairwise t-tests on these GIST features for all category combinations. We computed spatial frequencies at three energy levels (30%, 60%, and 90%), corresponding to the percentage of total spectral energy captured within the image sets. The statistical analysis revealed no significant differences (all pFDRs > 0.05) in the low-level features across categories (See Supplementary Fig. S3).

We partially occluded these scene images by placing a uniform grey rectangle over the lower right image quadrant (Fig. 2A–C, Smith and Muckli, 2010; Muckli et al., 2015; Revina et al., 2018; Morgan et al., 2019; Petro et al., 2023). We used a retinotopic mapping procedure to localise the mask location. This design allows us to localise the region of the retinotopic V1 cortex that responds to the occluded quadrant and receives informative bottom-up information at a different time than contextual feedback processing of the surrounding scene information. In previous studies, we did not present any scene information in the occluded part and were able to identify contextual feedback from other cortical regions. Here, we measure scene-specific activation in this masked region of V1 as it interacts with top-down activation at different time points. If contextual information is presented before the masked region, we can test how consistency influences later processing in the occluded area. Likewise, if no scene-specific context is presented before the bottom-up information, we can see how the contextual processing of scene information influences the BOLD signal after its initial processing in the target region (Fig. 2). The full stimulus image spanned 19.5 × 14.7° of visual angle with the grey occluder occupying ∼9 × 7°. We presented a centralised fixation with a black cross (0.1 × 0.1°) in the centre of the scene images.

### Experimental conditions

As referred to above, we distinguish two portions of our image stimuli: the ‘Surround’ and the ‘Target’, from which we derive five experimental conditions. The Surround refers to the three quarters of the scene that do not cover the lower right image quadrant (Fig. 2A). The Target refers to the lower right quadrant, which either contains scene information (as in the ‘Target only’ condition) or contains a grey occluder. Across our conditions, the Surround was presented before or after the Target, with the scene content between the Surround and Target either matching or not. Within the 2.6s of a single trial (the interval between trials was jittered, see below), the Target was always shown between 800ms and 1800ms from the trial start. In the Target-only condition, this was the whole trial. We presented the Surround from 0ms to 1000ms in the ‘before’ conditions. As a control of the causality of the top-down processes involved, we also tested the effect of presenting consistent and inconsistent contextual information after the Target, so from 1600ms to 2600ms in the ‘after’ conditions. Finally, the Surround was either consistent or inconsistent with the Target (i.e. from the same scene or not), yielding our 5 conditions (Fig. 2B).

### Mapping nonstimulated V1 - region of interest

Ten times per run, participants viewed high-contrast flickering checkerboards (4 Hz) at one of three visual locations, one at the lower right image quadrant (Target mapping), one over the image region not containing the Target (Surround mapping), and one at the border between the Target and Surround (Border mapping), Fig. 2C. The Target and Surround stimuli occupied the region of 9 × 7° visual angle and the region of 19.5 × 14.7° visual angle, while the border regions were mapped between the target and surround (approximately 0.5 diagonally from fixation) to minimise feedforward signal spillover, we independently mapped the border of the occluded region and excluded border-responsive voxels from the feedback ROI (Smith and Muckli, 2010; Morgan et al., 2019) (Fig. 2C). Mapping trials were used to identify the cortical representation of the occluded region in early visual cortex.

### Task and procedure

We designed an event-related fMRI paradigm consisting of the 5 experimental conditions (consistent surround before, inconsistent surround before, consistent surround after, inconsistent surround after, and target only) and 1 baseline condition. We designed our experimental stimulation using PsychoPy software (Peirce et al., 2019; Version 3; https://www.psychopy.org). We presented visual stimulation on a projector with a 60 Hz refresh rate. A tilted adjustable mirror installed on the head coil allowed participants to view the stimuli during the experiment, using a rear-projection screen (1024 x 768 resolution, 60Hz refresh rate). The main fMRI experiment consisted of three experimental runs. Each run contained 60 main and 10 mapping trials (4 for target mapping, 4 for border mapping, and 2 for surround mapping) and lasted approximately 15 min. The main trials consisted of a combination of 2.6s stimulus presentation and an average of 7.5s inter-stimulus interval (ISI), jittered between 6s and 9s.

To reduce perceptual adaptation to the scenes, we used a balanced Latin square design to derive the presentation order of 5 trial types, and we randomly assigned the five experimental conditions to these trial types at the start of each run. As a further control, we ensured that each condition (e.g. “consistent before”, “target only”, “inconsistent after”) in a 5-trial sequence was associated with images from all scene categories. In the “inconsistent before” and “inconsistent after” conditions, the contextual images were selected from non-presented surrounds from the 5-trial sequence. To ensure fixation, participants were required to press a button to respond to random colour changes of the central fixation cross, which could occur at any time within every minute with an interval of at least 20s between each change. Mapping trials occurred at the end of each group of 5 main trials. They consisted of a 12s contrast-reversing flickering (4Hz) checkerboard and a 12s fixation followed by the normal ISI jittered around 7.5s, resulting in an overall interval jittered between 18 and 21s after mapping trials.

We presented a retinotopic mapping run after the experimental runs to allow us to localise the boundaries of early visual areas V1-V3. The mapping stimulus was a combination of polar-angle and eccentricity high-contrast flickering checkerboards (Fig. 2B and Supplementary Fig. S1). As for the main runs, we engaged the participants to focus on the central dot with a task of spotting changes in fixation colour.

### MRI acquisition

We collected MRI data at the Centre for Cognitive Neuroimaging, University of Glasgow. We measured blood oxygen level-dependent (BOLD) signals by using a 3T Siemens Tim Trio MRI system with a 32-channel head coil and integrated parallel imaging techniques (IPAT factor: 2). Functional scanning used an echo-planar imaging sequence [echo time (TE): 30ms, repetition time (TR): 1500ms, the field of view (FOV): 200 mm, flip angle: 77°, 22 slices, spatial resolution: 2 x 2 × 2 mm] to acquire partial brain slices aligned to maximise coverage of early visual areas. We collected data from two main experimental runs for two participants in the pilot phase and three runs for the remaining 21 participants, one run of retinotopic mapping (combination of polar angle with eccentricity) and a 3D high-resolution anatomical scan [3D Magnetization Prepared Rapid Gradient Echo (MPRAGE), 1× 1 × 1 mm resolution, 192 slices] was performed in each scanning session.

### MRI data analysis

We analysed anatomical and functional MRI data using BrainVoyager (version 22.2, Brain Innovation, Maastricht, Netherlands). After brain extraction and inhomogeneity correction, we aligned anatomical data to the AC-PC plane and transformed it into Talairach space (Talairach and Tournoux, 1988). We pre-processed functional data using 3D motion correction, slice-scan-time correction, and a high-pass filter with a cycle of 5 sines/cosines (the value in retinotopic mapping data is 7). We then co-registered anatomical and functional data using boundary-based registration (BBR). BBR uses the WM/GM borders to align functional and anatomical data by maximising the boundaries between the tissue types (Greve and Fischl, 2009). This allowed us to create high-resolution volume time courses on which to perform statistical analyses. We generated an inflated 3D mesh cortical representation of the anatomical data to help us visualise functional activity and define the Regions of Interest (ROIs). To define individual subjects’ ROIs, we first delineated boundaries of the early visual cortex, including V1, V2, and V3, by using linear cross-correlation of eight polar angles from retinotopic mapping runs (Fig. 2B, second column, bottom row). Then, we ran a general linear model (GLM) to find the voxels within these regions with activations corresponding to the contrast “target > border and target > surround” in the mapping blocks (Fig. 2C, second column, top row). These contrasts ensured that selected voxels responded specifically to the Target region while excluding voxels responsive to the Surround and Border regions. This procedure removes voxels potentially influenced by surround stimulation and minimises lateral interactions from the border representation, thereby isolating voxels corresponding to the occluded Target region in V1. The results from retinotopic mapping allow us to find the active areas corresponding to the lower right target area in V1, and we used MATLAB to take the subset of voxels that have a t-value > 1 for the GLM contrast target minus surround as the final step of ROI definition (see Fig. 2C, right).

We then applied an ROI-wise deconvolution analysis for rapid event-related designs (Hinrichs et al., 2000) to the main trial data. Experimental protocol files defined the predictors corresponding to all conditions (trials) to a series of 20 time points (1.5s per point) across the haemodynamic response with the stimulus onset. To understand the BOLD signal change at a specific time after a stimulus, we fitted a 20-point deconvolution general linear model (deconvolution point 0 = stimulus onset) to estimate the haemodynamic response function for each predictor. These haemodynamic responses were deconvolved to give a BOLD response amplitude value in the form of beta-weighted estimates for each condition at each time point. We defined contrasts for response amplitudes between conditions of interest, allowing us to compare the effect of contextual consistency on the univariate BOLD response amplitude in the Target region of V1 (i.e. responding to the lower right image quadrant). We did not apply serial correlation correction because we found that it introduced a shift in the first deconvolution point (p < 0.1 for the difference between consistent before and inconsistent before) that was unrelated to any stimulation. We selected deconvolution points (D3, D4, D5, D6) containing the peak of the signal (%- transform) for both the Target only and ‘before’ conditions as well as the time-shifted ‘after’ conditions (Supplementary Fig. S2 shows individual data). False discovery rate (FDR) correction was applied across the seven planned contrasts, and corrected values are reported as ‘pFDR’.

To test whether increasing familiarity with the stimuli affected the BOLD response, we compared RUN1 and RUN3 separately for each condition using mean responses averaged across deconvolution points 3–6 and Welch's *t*-tests (FDR correction across all 5 conditions). No significant differences were observed in any condition (all pFDRs > 0.28), indicating no detectable effect of increasing stimulus familiarity on the BOLD response (Supplementary Fig. 4).
