# Supplementary material for: Context-sensitive V1 activity that depends on both coherence and surprise
Source: Curr Res Neurobiol. 2026 Sep 17;11:100168. doi: 10.1016/j.crneur.2026.100168 (PMC13632203; doi:10.1016/j.crneur.2026.100168)
Supplement: Supplementary file 1 [file mmc1.docx]

**SUPPLEMENTAL INFORMATION**

**Figure S1. Retinotopic and Checkerboard mapping experiment used to define participant-specific V1 regions of interest.**
A retinotopic and checkerboard mapping experiment was conducted for each participant to define the region of interest in the primary visual cortex. Left panel: retinotopic maps of early visual areas for each participant. The cortical representations corresponding to the lower-right target location in V1 are highlighted in red. These maps were derived using a GLM contrast comparing the target-mapping condition with the border- and surround-mapping conditions. Right panel: event-related BOLD signal changes during the mapping trials, shown from 0 to 20 s after stimulus onset. Error bars denote the standard error of the mean (SEM).

**Figure S2.** Individual data plots: Difference in mean BOLD signal changes between the conditions. The difference is calculated as the mean BOLD signal across deconvolution points 3, 4, 5, and 6 for each participant. **a.** Inconsistent before vs target only. **b.** Consistent before vs Target only. **c.** Inconsistent after vs Target only. **d.** Consistent after vs Target only. **e.** Inconsistent before vs Consistent before. **f.** Inconsistent after vs consistent after

**Figure S3. Low-level feature comparisons across image categories.** Low-level image properties were quantified using Python OpenCV and the MATLAB Natural Image Statistical Toolbox, including GIST descriptors, luminance, contrast, and spatial-frequency energy at 30%, 60%, and 90% levels. Pairwise comparisons between image categories were performed using independent-samples *t*-tests, and the resulting p-values were adjusted across comparisons using the false discovery rate procedure. Each matrix shows the FDR-corrected p-values for all pairwise category comparisons; diagonal cells are omitted. Panels are arranged as follows: top row, GIST descriptor (left), luminance (middle), and contrast (right); bottom row, 30% spatial frequency (left), 60% spatial frequency (middle), and 90% spatial frequency (right).

**Figure S4.Comparison of BOLD responses between RUN1 and RUN3.**Mean BOLD responses were averaged across deconvolution time points 3–6 and are shown separately for the consistent-after, consistent-before, inconsistent-after, inconsistent-before, and target-only conditions. Boxes indicate the interquartile range, horizontal lines indicate the median, whiskers extend to values within 1.5 times the interquartile range, and individual dots represent participant-level observations. RUN1 included 23 participants, and RUN3 included 21 participants. Differences between runs were assessed separately for each condition using two-sided independent-samples Welch’s t-tests. The reported p-values were adjusted across the five comparisons using the Benjamini–Hochberg false discovery rate procedure. The Welch t-statistic, degrees of freedom, and FDR-corrected p-value are displayed above each panel. Brackets indicate the RUN1–RUN3 comparison: n.s., pFDR ≥ .05; *, pFDR < .05; **, pFDR < .01; ***, pFDR < .001.
